# Two-stimuli manipulation of a biological motor

**DOI:** 10.1186/1477-3155-7-3

**Published:** 2009-05-15

**Authors:** Zorica Ristic, Marco Vitali, Alessandro Duci, Christian Goetze, Klaus Kemnitz, Werner Zuschratter, Holger Lill, Dirk Bald

**Affiliations:** 1 Department of Molecular Cell Biology, VU University Amsterdam, Amsterdam, the Netherlands; 2 Leibniz-Institute for Neurobiology, Magdeburg, Germany; 3 arivis Multiple Imaging Tools, Rostock, Germany; 4 EuroPhoton, Berlin, Europhoton, Berlin, Germany; 5 Technical University Berlin, Germany

## Abstract

F_1_-ATPase is an enzyme acting as a rotary nano-motor. During catalysis subunits of this enzyme complex rotate relative to other parts of the enzyme. Here we demonstrate that the combination of two input stimuli causes stop of motor rotation. Application of either individual stimulus did not significantly influence motor motion. These findings may contribute to the development of logic gates using single biological motor molecules.

## Findings

Biological nano-scale motors fulfil a broad range of tasks in living cells. Some motors like myosin, kinesin and dynein move in linear fashion. Other motors perform rotary motion, e.g. the bacterial flagellar motor or the enzyme F_1_-ATPase. F_1_-ATPase hydrolyses ATP into ADP and inorganic phosphate. It is the smallest biological rotary motor known, with a total molecular mass of ~400 kDa and the core subunits α_3_β_3_γ [1-3]. During enzymatic catalysis subunit γ rotates within the hexagonal α_3_β_3 _domain. This rotary movement has been microscopically monitored by attachment of large probes such as fluorescently labelled actin filaments and polymer microspheres to subunit γ [4-7]. In addition to plain motor observation, also manipulation of motor movement has been reported. Rotation in reverse direction was imposed on F_1_-ATPase using magnetic tweezers [8,9]. Furthermore, rotor movement was successfully modulated by chemical signals, including redox-switching [10,11], builtin Zn-sensitive switches [12], small organic molecules [13-15] as well as by temperature control [16,17]. However, these experiments describe the response of F_1 _to individual stimuli and do not reveal how simultaneously acting stimuli are processed by the motor.

Here we report manipulation of the F_1_-ATPase motor movement at single molecule level by concerted optical and chemical input stimuli. We combined an optical stimulus (high-intensity illumination) with a chemical stimulus (rhodamine 6G), on the rotary movement of single F_1 _molecules.

Biotin-PEAC maleimide was purchased from Dojindo (Kumamoto, Japan). Streptavidin-coated microspheres (mean diameter: 510 nm) were from Bangs Laboratories, Inc. (Fishers, Indiana, USA). Other chemicals were of the highest grade commercially available.

### Preparation of F1-ATPase

The α_3_β_3_γ core complex of F_1_-ATPase originating from *Bacillus *PS3 was prepared as previously described in [10] and hereinafter referred to as F_1_-ATPase. The enzyme was over-expressed in *Escherichia coli *strain JM103 uncB-D using the pkkHC5 expression plasmid [10]. This plasmid codes for the α, β, and γ subunits of the thermophilic *Bacillus *PS3 F1-ATPase, carrying a decahistidine tag at the N terminus of the β subunit and the mutation γSer106→Cys.

### Rotation Assay

F_1_-ATPase was biotinylated at a single cysteine residue in subunit γ using biotin-(PEAC)5-maleimide (Dojindo, Japan), as described elsewhere [10]. The biotinylated F_1_-ATPase (30 nM) in an assay mixture containing 10 mM 3-(N-Morpholino) propanesulfonic acid (MOPS)/KOH (pH 7.0), 50 mM KCl and 2 mM MgCl_2 _(buffer A) was infused into a flow cell, constructed from microscope cover slips as described [4], and incubated for 5 min to allow for immobilization. The flow cell was washed with 100 μl of Buffer A supplemented with 10 mg/ml bovine serum albumin (buffer B). Subsequently, a suspension of streptavidin-coated polystyrene beads (Bangs Laboratories, diameter 510 nm) suspended in Buffer B was infused and incubated for 15 min. Next, 100 μl of reaction buffer (Buffer B supplemented with 2 mM ATP, 4 mM MgCl_2_, 2.5 mM phosphoenolpyruvate, and 0.1 mg/ml pyruvate kinase (Roche Applied Science) in the absence or in the presence 100 μM Rhodamine 6G (Merck) was infused and microscopic observation was started. Rotation of beads was observed under bright field illumination with an inverted fluorescence microscope (TI Eclipse, Nikon) equipped by a Nikon Plan. Apo. 100× (N.A. 1.4) objective. Images were recorded with an Andor iXon DU-897BI EMCCD camera (Andor Technology, Belfast, UK) at 25 Hz frame rate. Image analysis was done using self made tracking routines under Matlab (The MathWorks, Natick, USA) and the open-source image analysis software ImageJ. Bright field illumination was performed by an attenuated 100W Halogen lamp (35 mW/cm/^2 ^on the sample).

High illumination intensity of the probe was performed by 110 W Mercury lamp in epi-fluorescence illumination. The excitation wavelength was selected by a 540 ± 10 nm interference filter.

### Motor movement in absence of input stimuli

**Figure 1:**
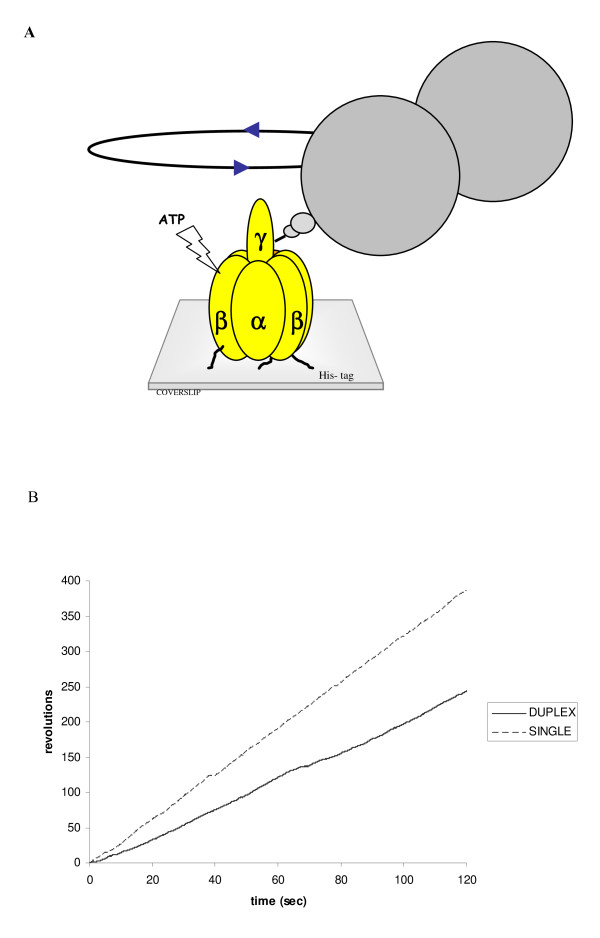
**Rotary movement of F_1_-ATPase motor**. (A) Schematic view of the experimental system for the observation of F_1_-ATPase rotation [7,11]. The polystyrene bead (diameter 0.51 μm) is connected to the F1 motor (not to scale). (B) Time course of F_1_-ATPase rotation. Typical traces for single beads (dashed line) and duplex beads (straight line) bound to one F_1_-ATPase molecule are shown.

ATP-driven rotation of F_1_-ATPase subunit γ was visualized by attachment of a bead to the γ subunit (Fig. 1a) [7,11], typical time courses of the rotational movement of two molecules F_1 _are shown in Fig. 1b. Rotation of both single-bead as well as duplex-beads was unidirectional, continuous and directions were always counter-clockwise when viewed from top (Fig. 1b, [6]). Bead rotation occasionally displayed pauses and subsequently resumed rotation. These pauses have been described previously and may be attributed to transient inhibition of F_1 _by Mg-ADP [18,19].

### Motor response to concerted chemical and physical input

**Figure 2:**
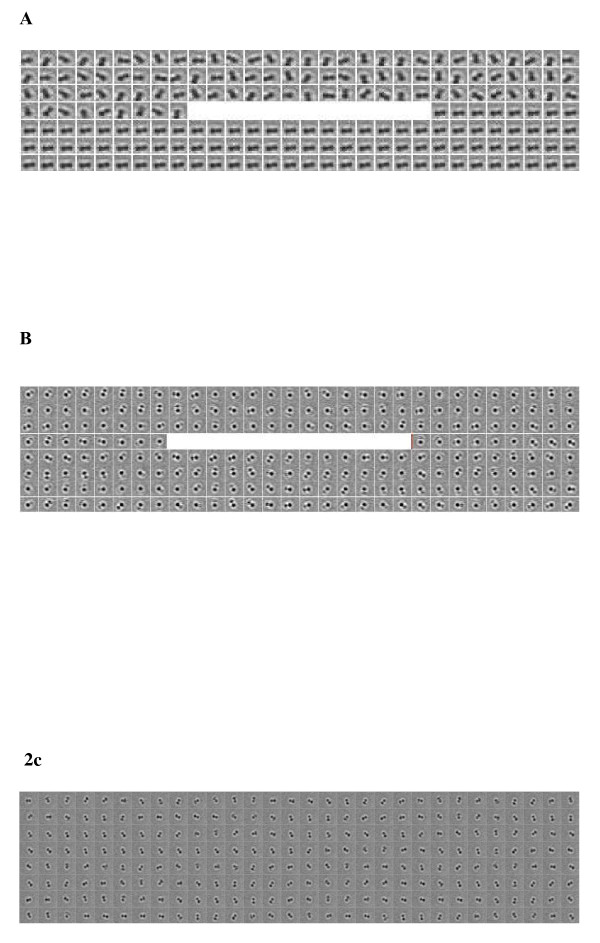
**Manipulation of F_1 _rotor motion by optical and chemical inputs**. Sequential images of a rotating beads before and after a pulse (10 sec) of high intensity white light illumination (white bar) in the presence (A) or absence (B) of rhodamine 6G. (C) Rotating beads in the presence of rhodamine 6G, but without light pulse.

Next, we determined the motor response to concerted physical and chemical stimuli. Illumination of the samples with light at 540 ± 10 nm for 5–10 sec at maximum intensity (110 W/cm^2^) in the presence of rhodamine 6G lead to a complete arrest of motor movement within the duration of the light pulse (Fig. 2a). This light-induced motor response was highly reproducible and observed for >90% of all investigated motor molecules (n = 20), with "motor arrest" defined as <1 revolution *per *minute of a single or a duplex bead. These results indicate that rotation of the F_1_-motor can be stopped by the combination of an optical and a chemical input signal.

### Motor response to individual input variables

We have observed a dramatic response of F_1_-ATPase motor movement to two combined inputs. Next, we assessed the two inputs imposed separately on the rotating motor. Firstly we tested the effect of high light intensity on F_1 _rotation in the absence of rhodamine 6G. Typically, no significant effect on motor movement was detected (Fig. 2b), only <10% of the observed F_1 _– ATPase molecules (n = 22) stopped upon illumination.

Subsequently we evaluated the effect of the chemical input (rhodamine 6G) alone on motor movement. As depicted in Fig. 2b, rhodamine 6G alone did not significantly influence motor rotation (<10% of n = 20 observed molecules arrested). Turnover of ATP by F_1_-ATPase in bulk-phase is influenced by rhodamine 6G and related lipophilic cations [20-28]. Whereas, low concentrations of rhodamine stimulate F_1_-ATPase (up to 10 μM), higher concentrations lead to enzyme inhibition [20,21]. Rhodamine 6G at higher concentration is believed to bind F_1_-ATPase at least at two binding sites [20-28]. High intensity illumination may cause photoreactions that modulate the affinity of rhodamine 6G for F_1_-ATPase [29-31].

We have demonstrated that the movement of a biological motor can be arrested by synergistic inputs of optical and chemical stimuli. Motor arrest is observed at single molecule level and does not occur when the input stimuli are applied separately. The motor response reported here is is consistent with a function as an "AND" logic gate in terms of producing a single output on two concerted inputs [32-34]. For full implementation of a motor protein "AND" gate, reversibility of the motor system response is an important factor. Experiments to gain a deeper understanding of the response mechanism and to improve reversibility are on-going in our laboratory. Biomolecules acting as "AND" gates in bulk-phase have been described earlier, e.g. light dependent release of an unfolded fluorescent protein from a chaperone protein [34], or an enzyme-based logic gate [35]. Extending the work of these authors, our results may help to develop motor protein-based logic gates, operating and monitored at the single molecule level.

## Competing interests

The authors declare that they have no competing interests.

## Authors' contributions

ZR performed motor labelling and microscopic observation, MV prepared the microscope set-up and took images, AD and CG carried out image analysis, KK, WZ HL and DB conceived the experiments, DB coordinated the study. All authors read and approved the final manuscript.
